# Mechanisms and regulation of neurotrophin synthesis and secretion

**DOI:** 10.17712/nsj.2016.4.20160080

**Published:** 2016-10

**Authors:** Mohammad A. Al-Qudah, Ahmed Al-Dwairi

**Affiliations:** *From the Department of Physiology (Al-Qudah, Al-Dwairi), Jordan University of Science and Technology, Irbid, Jordan, and the Department of Physiology and Biophysics (Al-Qudah), VCU Program in Enteric Neuromuscular Sciences, School of Medicine, Virginia Commonwealth University, Richmond, Virginia, United States of American*

## Abstract

Neurotrophins are secreted proteins that are synthesized as pre-pro-neurotrophins on the rough endoplasmic reticulum, which are subsequently processed and then secreted as mature proteins. During synthesis, neurotrophins are sorted in the trans-Golgi apparatus into 2 pathways of secretion; the constitutive and the regulated pathways. Neurotrophins in the constitutive pathway are secreted cautiously without any trigger, while in the regulated pathway of secretion an external stimulus elevates the calcium concentration intracellularly leading to neurotrophin release. The regulation of sorting and secretion of neurotrophins is critical for several processes in the body, such as synaptic plasticity, neurodegenerative disorders, demyelination disease, and inflammation. The purpose of this review is to summarize the current mechanisms of neurotrophin sorting and secretion.

Neurotrophins are structurally related dimeric proteins that were originally viewed as neuronal survival factors released from target tissues. To date, they are critical players in many facets of the central and peripheral nervous systems functions, such as regulation of neuronal differentiation, proliferation, migration, and activity-dependent synaptic plasticity. Four members of the mammalian neurotrophins family have been identified; nerve growth factor (NGF), brain derived neurotrophic factor (BDNF), neurotropin-3 (NT-3), and neurotrophin-4/5 (NT-4/5). These members share a number of structural and chemical properties, including more than 50% sequence homologies in the primary structure, approximately similar molecular weight, 3 disulfide bonds that form a cysteine knot and isoelectric points ranging from 9-10.^1^-^4^ Neurotrophins mediate their physiological functions by binding to 2 distinct classes of cell surface receptors. The first class includes the 3 members of the tropomyosin-related kinase (Trk) family of receptor tyrosine kinases. The Trk receptor has 3 domains, an extracellular domain, a single transmembrane domain, and an intracellular domain that contains the catalytic tyrosine kinase domain. A specific Trk receptor binds to each neurotrophin family member with high affinity, where NGF interacts with Trk A, BDNF and NT-4 interact with Trk B, and NT-3 interacts with Trk C. The NT-3 also binds to Trk A and Trk B, but with less affinity. Only mature neurotrophins activate the Trk receptors and 3 major pathways result from this activation, the small Guanosine Triphosphatase (GTPase); Ras, phosphatidylinositol 3-kinase, and phospholipase C-g1(PLC-g1), and their downstream cascading proteins. Ras activation controls normal survival and differentiation by activating mitogen-activated protein kinase (MAPK) cascades. Phosphatidylinositol 3-kinase governs several neuronal functions such as survival and neurite out growth through activation of the protein kinase B commonly known as AKT Lastly, PLC-g1 activation accounts mainly for the control of activity-dependent synaptic plasticity.^1^,^4^,^5^ The second class of neurotrophin receptor is the P75 neurotrophin receptor (P75NTR), which belongs to the tumor necrosis superfamily. The P75NTR contains an extracellular domain that consists of 4 cysteine-rich regions, a single transmembrane domain, and a death cytoplasmic domain. All neurotrophins interact with P75NTR with the same low affinity. The P75NTR influences the affinity and the specificity of neurotrophins to Trk receptors by forming heterodimers with Trk receptors.^1^,^5^,^6^

Characterizing the intracellular biosynthesis and sorting of neurotrophins within a cell is an important aspect of understanding how neurotrophins are released and function, as it is critical for several physiological and pathophysiological processes such as synaptic plasticity, memory formation, neurodegenerative disorders, and demyelination diseases and inflammation. The purpose of this review is to summarize the current knowledge of the mechanisms regulating neurotrophins sorting and secretion. Most of the results reviewed here were obtained from studies conducted either on cultured neurons expressing endogenous/overexpressing neurotrophins or on neuroendocrine cell lines pheochromocytoma cell line PC-12, pituitary cell line AtT20, and fibroblast cell lines; COS 1 and COS 7 (PC12, AtT20, COS1, and COS7). However, secretion of neurotrophins from non-neuronal cells will also be reviewed.

## Synthesis of neurotrophins

**Figure 1:**
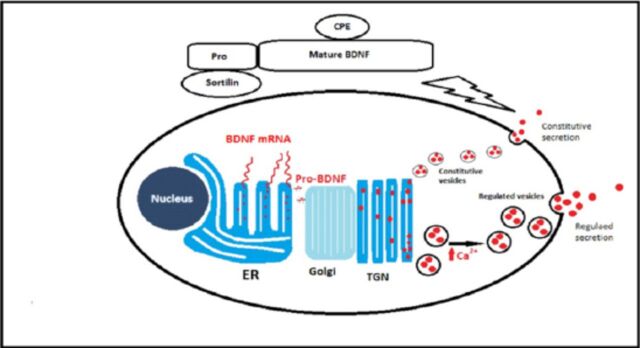
Neurotrophin synthesis and sorting. Neurotrophins are synthesized as pre-pro-neurotrophins on the rough endoplasmic reticulum (ER) and then move to the golgi apparatus to accumulate in the trans-Golgi network (TGN). In the TGN, neurotrophins are sorted to either the constitutive secretory vesicles or the regulated secretory granules. Sortilin and carboxypeptidase E (CPE) are important sorting receptors that mediate neurotrophins targeting to the regulated secretory granules. BDNF - brain derived neurotrophic factor, mRNA - messenger ribonucleic acid

As secreted proteins, all neurotrophins are synthesized as pre-pro-neurotrophins on the rough endoplasmic reticulum (ER).^7^-^9^ The pre-sequence functions to direct nascent neurotrophins polypeptide synthesis to the rough ER, and once inside the ER the pre-sequence is cleaved off immediately and the pro-neurotrophins can dimerize into homodimers and to a lesser extent heterodimers. Post-translational modifications can also take place while the pro-neurotrophins are translocated from Golgi apparatus to the trans-Golgi network (TGN).^9^ These modifications are N-linked glycosylation in the pro-domain, sulfation of these N-linked oligosaccharides and cleaving of the pro-domain to yield the mature neurotrophins. Once neurotrophins reach the TGN, an important sorting mechanism will take place. Two discrete types of secretory vesicle are produced and filled with neurotrophins based on the mechanism of their secretion. The first type of vesicles are small-diameter granules (50-100 nm) and are continuously released in a calcium^2+^ (Ca^2+^) independent fashion in the absence of any triggering stimulus. This pathway represents the constitute pathway of secretion. While in the second type that represents the regulated pathway of secretion, the vesicles are larger (100-300 nm), bud from the TGN, and fuse with plasma membrane only in a Ca^+2^ dependent manner in response to an extracellular triggering event (**Figure 1**).^9^

## General concept

Neurotrophin sorting into either of the pathways of secretion depends on several factors, such as the localization of protein convertases (PCs) and their pH-optimum in different compartments of the cells, conserved sequences in the pro and mature domains, post translational modifications, and sorting receptors.^9^,^10^ The PCs are a family of serine endoproteases, which are members of the subtilisin super family that activate protein precursors. They include, furin, PC1/PC3, PC2, PC4, PACE5, PC5/PC6, PC7/LPC, PC8, and the mammalian pyrolysin-like enzyme known as SKI-1/S1P.^11^,^12^ The PCs cleave precursor proteins behind a pair of basic residues Arginine Arginine or Lysine Arginine. Different PCs have specific efficiency to cleave different precursor proteins. Furin, PC5-B, PC7, PC4, PACE4, PC5, and SKI-1 are the convertases involved in the processing of neurotrophins secreted via the constitutive pathway. Conversely, PC1 and PC2 are the only members of the mammalian PCs found in the dense core vesicles secreted by the regulated pathway of secretion. Moreover, the processing efficiency of neurotrophins depends in part on the last half of the pro-region of these proteins.^13^ It has been reported that sequence information in the pro-domain is necessary to target neurotrophins to the regulated secretory vesicles.^10^,^14^ Sortilin, which is an important intracellular sorting receptor, plays a role in targeting some neurotrophins to the regulated secretory vesicles via interaction with a specific sequence of the pro-domain of neurotrophins.^15^-^17^ In addition, to sequence information in the pro-region of neurotrophins, the mature neurotrophin has a sequence signal that is recognized by the sorting receptor carboxypeptidase E (CPE) and results in targeting the neurotrophins to the regulated pathway of secretion.^18^,^19^ Finally, the importance of these sorting motifs in targeting neurotrophins to the secretory vesicles has been confirmed by experiments, which showed that swapping of these motifs between different neurotrophins might alter the sorting of neurotrophins to either of the secretory pathways.^9^

## Brain derived neurotrophic factor processing and targeting

Compared to other neurotrophins, BDNF is targeted to the regulated secretory vesicles most efficiently.^9^,^20^ Many explanations have been documented for this efficient targeting to the regulated pathway of secretion. In TGN, BDNF is less sensitive to cleavage by PCs specific for the constitutive secretion, especially furin.^10^ Moreover, a valine-to methionine (Val66Met) single-nucleotide polymorphism in the pro-BDNF gene impairs the regulated secretion of BDNF.^21^,^22^ It was found that a targeting signal in the pro-domain of BDNF surrounding the methionine substitution is involved in the efficient sorting to the regulated secretory vesicles. Furthermore, the amino acid residues encompassing the met substitution represent a binding site for the intraluminal domain of the Golgi resident sorting receptor sortilin. The interactions between sortilin and these residues accounts for the optimal trafficking of BDNF to the regulated pathway of secretion.^23^ In addition to the sorting domain in the pro-BDNF, the mature BDNF sequence also influences sorting. The tertiary structure of the mature BDNF contains a conserved sorting motif consisting of (IIe16, Glu18, IIe105, and Asp106) with their side chains accessible on the protein surface. The electrostatic binding of the acidic residues of this sorting motif with the basic residues of the sorting receptor CPE ensures BDNF sorting to the regulated secretory pathway.^24^,^25^ The importance of this sorting mechanism in directing BDNF to the regulated pathway of secretion was tested via measuring BDNF secretion in cells harboring the wild type pro-BDNF and other cells infected with pro-BDNF, in which the acidic residues in the sorting motif were mutated to hydrophobic residues.^19^ The mutated pro-BDNF in that study resulted in miss sorting of BDNF to the permissive pathway of secretion, and when all the 4 amino acids in the sorting signal were mutated; the regulated secretion of BDNF was abolished. Moreover, the same lab showed that BDNF secretion via the regulated pathway was obliterated in neuronal cells from CPE-/-. Notably, the addition of this sorting motif to NGF, which lacks a similar motif and is secreted largely by the constitutive pathway, redirected NGF significantly to the regulated secretory vesicles.^26^ These results together indicate that targeting of BDNF to the regulated secretory granules involves a receptor-mediated mechanism, similar to the mechanism involved in the sorting of pro-insulin and pro-opimelanocortin.^16^

## Neurotropin-3 targeting and processing

Like BDNF, NT-3 is targeted to the regulated pathway of secretion.^9^,^27^ However, other studies have reported that NT-3 is targeted mainly to the constitutive pathway of secretion and could be only targeted to the regulated pathway when coexpressed with BDNF by forming hetrodimers.^28^ Unlike BDNF, the sorting receptors, the targeting sequences, and the molecular mechanisms underlying the regulated secretion of NT-3 are still largely undetermined.

## Processing of nerve growth factor and neurotropic-4

Both NGF and NT-4 are primarily sorted to the constitutive secretory vesicles.^9^,^26^ However, it has been documented that those neurotrophins, especially NGF, are secreted via the regulated pathway.^9^,^29^ The regulated secretion of NGF was claimed to be a result of its overexpression in cultured neurons and cell lines.^30^,^31^ It was also reported that coexpression of BDNF and NGF resulted in the formation of heterodimers, suggesting that BDNF sorting in these heterodimers may influence the targeting of NGF that is usually secreted via the constitutive pathway.^9^,^31^ In contrast to this observation, heterodimers of NT-4 (which is secreted preferentially via the constitutive pathway) and BDNF are secreted constitutively; in this case NT-4 redirects BDNF secretion from the regulated to the constitutive pathway.^3^

Nerve growth factor is cleaved efficiently by furin, PACE4, and PC5/6B, which are all present in the constitutive pathway of secretion.^9^ Intriguingly, a recent study found that furin cleavage of pro-NGF at the consensus sequence arginine-serine-lysine-argenine seems to be obligatory for NGF secretion by the regulated pathway.^32^ These results suggest that neurotrophin targeting depends on the presence of a specific pro-convertase in a given cell type by acting as a sorting receptor and controlling the amount of mature neurotrophins in the TGN. In this scenario, it could be assumed that uncleaved pro-NGF or pro-NT-4 can be targeted to the constitutive secretory vesicles, while mature forms are targeted to the regulated pathway of secretion.^33^

## Neurotrophin secretion

**Figure 2:**
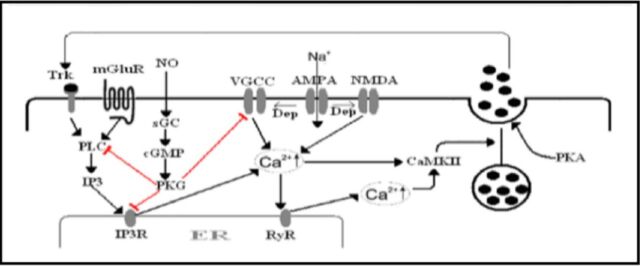
The mechanisms of regulated neurotrophin secretion. Neurotrophins secretion relies on elevation of intracellular Ca^2+^ concentration. The Ca^2+^ influx through voltage gated calcium channels (VGCC) and/or NMDA receptor upon depolarization and Ca^2+^ release from IP3R in response to metabotropic glutamate receptor (mGLUR) activation causes an increase in intracellular Ca^2+^. The Ca^2+^ influx induces Ca^2+^ release from ryanodine receptors (RyR), which amplifies the CA^2+^ rise. The Ca^2+^ activates calcium calmodulin kinase II (CaMKII) inducing the fusion of the secretory vesicles in a mechanism involving the protein kinase A (PKA) action. Secreted neurotrophins act in a positive feedback mechanism to augment intracellular Ca^2+^ rise. Activation of PKG via the nitric oxide (NO) signaling pathway down-regulate neurotrophins secretion by desensitizing many proteins involved in the release process. Trk - tropomyosin-related kinase, PLC -phospholypase C, IP3- inositol 1, 4, 5-trisphosphate, IP3R- IP3-receptor, sGC- soluble guanylyl cyclase, PKG- protein kinase G, Na+ - Sodium, AMPA-a-amino-3-hydroxy-5-methyl-4-isoxazolepropionic acid receptor, NMDA - The N-methyl-D-aspartate receptor, Dep - depolarization, Ca^2+^ - calcium^2+^

Several studies have been conducted to address neurotrophin secretion using mainly 2 approaches. The first approach utilizes enzyme-linked immunosorbent assay and/or Western blot to measure BDNF secretion into the extracellular medium. In this approach, either endogenous or overexpressed BDNF could be measured. The BDNF tagged with green fluorescent protein (GFP) is the second approach, which has been used to measure BDNF secretion from cultured neurons. This approach allows direct visualization of BDNF-GFP secretion as a decrease of intracellular fluorescent intensity. Both neuronal cultures and neuroendocrine cell lines were used to investigate secretion of neurotrophins.^34^ **Figure 2** depicts a scheme of different signaling cascades involved in the regulated secretion of neurotrophins.

## Brain derived neurotrophic factor secretion

The regulated secretion of BDNF has attracted the interest of many investigators. High potassium (K^+^) and cyclic adenosine monophosphate (cAMP) induced the secretion of BDNF,^19^,^35^ and this secretion was dependent on extracellular Ca^2+^.^19^ Additional studies showed that BDNF secretion in response to both 50 mM K^+^ and glutamate was dependent on intact intracellular Ca^2+^ stores as the intracellular high affinity calcium chelator 1,2-bis (2-aminophenoxy) ethane-N,N,N¢,N¢-tetraacetic acid pentaacetoxymethyl ester abolished the depolarization-mediated BDNF secretion.^9^ The secretion of BDNF by glutamate in the absence of extracellular Ca^2+^ may be explained by the activation of metabotropic glutamate receptors that leads to the generation of inositol 1, 4, 5-trisphosphate (IP3) which activates Ca^2+^ channels on the ER. Activation of Ca^2+^ channels causes an increase in intracellular Ca^2+^ and eventually BDNF release.^36^,^37^ Consistent with these results, glutamate-induced BDNF secretion was shown to be dependent on intracellular Ca^2+^ in oligodendrocytes.^37^ In the same study, BDNF secretion was solely dependent on metabotropic glutamate receptors activation because neither N-methyl-D-aspartate receptor (NMDA) nor kainite was capable of stimulating the release of BDNF. On the other hand, BDNF secretion via high K^+^ induced depolarization cannot be explained in the absence of extracellular Ca^2+^^38^ because it is well documented that high K^+^ activated Ca^2+^ influx via the voltage gated Ca^2+^ channel (VGCC) requires extracellular Ca^2+^, and it is the major mechanism responsible for vascular release.^39^

Glutamate mediated-secretion of BDNF has been demonstrated to take place via a-amino-3-hydroxy-5-methyl-4-isoxazolepropionic acid (AMPA) receptors, in addition to metabotropic glutamate receptors.^37^ In this respect, AMPA application can activate inotropic glutamate receptors causing depolarization-induced Ca^2+^ influx through activation of the VGCC.^9^ The importance of Ca^2+^ influx-mediated BDNF secretion was also confirmed by the absence of BDNF secretion in experimental conditions lacking extracellular Ca^2+^.^40^ The Ca^2+^ influx occurs through the VGCC in these experiments. Finally, depolarization-induced BDNF secretion was dependent on Ca^2+^ influx through N-methyl D-aspartate receptor or L-type VGCC, which further confirms the importance of extracellular Ca^2+^ involvement in BDNF secretion.^41^

Interestingly, BDNF secretion is stimulated by the activation of Trk receptors via neurotrophins.^42^ Exogenous application of NT-4/5- or NT-3 stimulated the secretion of BDNF via activation of Trk receptors but not P75 receptors, and the secretion was dependent on intracellular Ca^2+^.^37^ Nerve growth factor has been shown to stimulate BDNF secretion by activating both TrkA receptors and P75, in which inhibition of both receptors together was necessary to attenuate NGF-induced BDNF secretion. The signal transduction pathways for neurotrophins-induced BDNF secretion seem to involve neurotrophins activation of Trk receptors that in turn mediates PLC-g1 activation, and subsequently IP3-induced mobilization of Ca^2+^ from the ER. Ultimately, the secretion of BDNF due to the increase of intracellular Ca^2+^ takes place.^42^ In addition to the endogenous BDNF that is stored in post-Golgi granules, exocytosed BDNF can re-enter postsynaptic sites in the dendrite of cultured hippocampal neurons and is later secreted in an activity-dependent manner.^43^

It is noteworthy that BDNF secretion can also be induced by electrical stimulation (ES). This type of secretion depends on the pattern of ES where high frequency burst stimulation is capable of inducing the release, while a low frequency pattern is ineffective. The BDNF release via this mechanism is dependent on repetitive Ca^2+^ influx through activation of VGCC and/or NMDA. The Ca^2+^ influx induced Ca^2+^ mobilization from intracellular stores is also needed for long lasting stimulus-induced BDNF release. The elevation of intracellular Ca^2+^ activates calcium calmodulin kinase II that mediates secretory vesicles fusion with plasma membrane. Protein kinase A also plays a role in BDNF secretion as it seems to prime the secretory vesicles for secretion.^9^,^41^ Recently, supporting data^44^ showed that Schwan cells release BDNF in response to ES in calcium dependent mechanisms involve Ca^2+^ influx from VDCC and from internal stores. Moreover, calcium calmodulin dependent protein kinase IV, cAMP response element-binding protein, and MAPK were found to play important roles in the ES-induced BDNF release from Schwan cells.^44^

One significant finding demonstrated that BDNF regulated secretion was dependent on protein kinase C (PKC) activation. Treatment of microglial cells with ceramide enhances the secretion of BDNF by a mechanism that seems to involve PLC phosphorylation and subsequent activation of PKC.^45^ Another study demonstrated that phosphorylation of extracellular signal-regulated kinase (ERK) 1/2 is involved in BDNF secretion as inhibition of ERK by the specific MAP kinase inhibitor-U0126 inhibited the regulated secretion of BDNF in rat primary astroglia cultures.^46^

In the previous section we dealt with mechanisms that upregulate BDNF secretion, none of the above reviewed articles investigated how BDNF secretion can be down-regulated. Nitric oxide (NO) negatively regulates BDNF release by inhibiting intracellular Ca^2+^ mobilization. Nitric oxide synthase is activated via NO and results in the activation of soluble guanylyl cyclase that generates cyclic guanosine monophosphate from GTP, which in turn activates protein kinase G (PKG). Activated PKG phosphorylates many proteins that are responsible for the elevation of intracellular Ca^2+^ including IP3 receptors and PLC^47^ (**Figure 2**).

## Secretion of nerve growth factor and other neurotrophins

Nerve growth factor is primarily secreted spontaneously without any triggering stimulus. The basal secretion of NGF was inhibited by lowering the incubation temperature, which is a feature of the constitutive pathway of secretion. Moreover, inhibition of the action potential generation by tetrodotoxin resulted in partial blockade of basal NGF secretion. On the other hand, other investigators reported activity-dependent secretion of NGF following either high extracellular K^+^ or glutamate application.^9^ In a recent study addressing NGF sorting, high K^+^ medium stimulated NGF secretion only if the pro-NGF was cleaved correctly in the TGN.^48^

The secretion of NT-3 was reported in a few studies, high K^+^ induced depolarization stimulates NT-3 secretion,^9^ and neurotrophins induce the release of NT-3.^49^ Finally, the regulated secretion of NT-3 depends on Ca^2+^ influx through VGCC or NMDA receptors. The contribution of the internal stores to the initial Ca^2+^ influx seems to sustain the release process.^41^

## Secretion of neurotrophins from non-neuronal tissues

Neurotrophins are produced from non-neuronal cells in addition to neuronal cells. In the airways, neurotrophins are produced from structural cells (epithelial, fibroblast, and smooth muscle cells) and immune cells, and their production is influenced by the pathophysiological status of these cells.^50^ Furthermore, periodontal tissues synthesize and secrete neurotrophins, which might play a role in healing of these tissues.^51^ Neurotrophins, specifically BDNF, has been reported to be secreted from enteric neurons, enteroendocrine cells, and smooth muscle cells in the gastrointestinal tract.^52^

Both the constitutive and regulated pathways of neurotrophin secretion are reported in non-neuronal tissues. In the airways, BDNF and NGF are secreted constitutively from immune, epithelial, and smooth muscle cells.^50^,^52^ However, these cells can secrete neurotrophins in an activity-dependent manner. For example, BDNF is present in airway smooth muscle cells, and several stimuli can induce its release such as inflammatory cytokines, neurotransmitters, and sex hormones both under physiological and pathological conditions.^53^-^55^ The regulated secretion of neurotrophins in these cells is under the control of intracellular Ca^2+^^53^,^55^ and cAMP.^56^ Moreover, the Val66Met polymorphism in the BDNF gene has been linked to pathological conditions in the airways such as asthma;^57^,^58^ whether this polymorphism affects the secretion of BDNF as in neuronal cells needs further investigation.

Both BDNF and NGF are produced from smooth muscle cells of the bladder where they play several roles and interact with neurotransmitters such as pituitary adenylate cyclase activating peptide (PACAP) and VIP.^59^-^61^ Caffeine enhances NGF expression in all central micturition areas suggesting a regulated section pathway for NGF in bladder smooth muscle cells.^62^

Intestinal smooth muscle cells secret BDNF constitutively, and gut neuropeptides such as substance P (SP) and PACAP upregulate this secretion. Moreover, chelating intracellular calcium abolished the effect of SP and PACAP suggesting that BDNF is sorted to the regulated pathway of secretion in intestinal smooth muscle cells.^52^,^63^ The mechanisms that control BDNF synthesis and secretion need to be investigated in gut smooth muscle cells.

The survival of vascular endothelial and smooth muscle cells along with the cardiomyocytes requires the presence of neurotrophins.^4^,^64^ Neurotrophins control the processes of angiogenesis and vasculogenesis. However, few experiments have been carried out to study the regulation of neurotrophins synthesis and secretion in the cardiovascular system. In vascular smooth muscle cells, intact furin was essential for mature NGF secretion and for the survival of developing smooth muscle cells.^11^ However, sortilin was essential for pro-NGF secretion and vascular smooth muscle apoptosis.^65^

In summary, most of the available data regarding neurotrophin synthesis and release is from studies on the nervous system. Such data suggest that secretion of neurotrophins is dependent on cell type and stimulus. As neurotrophins are secreted from non-neuronal cells as well and act in many other ways, it will be interesting to investigate the molecular mechanisms involved in the secretion of neurotrophins in non-neuronal cells for a better understanding of their functions outside the nervous system.
